# Eye movement biomarkers allow for the definition of phenotypes in Gaucher Disease

**DOI:** 10.1186/s13023-020-01637-9

**Published:** 2020-12-17

**Authors:** Aimee Donald, Chong Y. Tan, Anupam Chakrapani, Derralyn A. Hughes, Reena Sharma, Duncan Cole, Stanislav Bardins, Martin Gorges, Simon A. Jones, Erich Schneider

**Affiliations:** 1 Manchester Centre for Genomic Medicine, St Mary’s Hospital, Manchester, UK; 2 Addenbrooke’s Hospital, Cambridge, UK; 3 Great Ormond Street Hospital, London, UK; 4 Royal Free Hospital, London, UK; 5 Salford Royal Foundation Trust, Salford, UK; 6 Cardiff and Vale University Health Board, Cardiff, Wales UK; 7 Ludwig-Maximillans-Univesitat Munchen, Munich, Germany; 8 Institute of Medical Technology, Brandenburg University of Technology Cottbus – Senftenberg, Cottbus, Germany

**Keywords:** Rare disease, Neuronopathic, Saccades, Video-oculography, Eye tracker, Ocular-motor, Neurodegenerative disease

## Abstract

**Background:**

Neurological forms of Gaucher disease, the inherited disorder of β-Glucosylceramidase caused by bi-allelic variants in *GBA1,* is a progressive disorder which lacks a disease-modifying therapy. Systemic manifestations of disease are effectively treated with enzyme replacement therapy, however, molecules which cross the blood–brain barrier are still under investigation. Clinical trials of such therapeutics require robust, reproducible clinical endpoints to demonstrate efficacy and clear phenotypic definitions to identify suitable patients for inclusion in trials. The single consistent clinical feature in all patients with neuronopathic disease is the presence of a supranuclear saccadic gaze palsy, in the presence of Gaucher disease this finding serves as diagnostic of ‘type 3’ Gaucher disease.

**Methods:**

We undertook a study to evaluate saccadic eye movements in Gaucher patients and to assess the role of the EyeSeeCam in measuring saccades. The EyeSeeCam is a video-oculography device which was used to run a protocol of saccade measures. We studied 39 patients with non-neurological Gaucher disease (type 1), 21 patients with type 3 (neurological) disease and a series of 35 healthy controls. Mean saccade parameters were compared across disease subgroups.

**Results:**

We confirmed the saccadic abnormality in patients with type 3 Gaucher disease and identified an unexpected subgroup of patients with type 1 Gaucher disease who demonstrated significant saccade parameter abnormalities. These patients also showed subtle neurological findings and shared a *GBA1* variant.

**Conclusions:**

This striking novel finding of a potentially attenuated type 3 Gaucher phenotype associated with a specific GBA1 variant and detectable saccadic abnormality prompts review of current disease classification. Further, this finding highlights the broad spectrum of neuronopathic Gaucher phenotypes relevant when designing inclusion criteria for clinical trials.

## Background

Gaucher Disease is a lysosomal storage disorder (LSD) resulting from deficiency of β-Glucosylceramidase (glucocerebrosidase), a lysosomal enzyme which hydrolyses the substrate glucosylceramide; a sphingolipid. Deficiency is secondary to recessively inherited mutations of the *GBA1* gene (OMIM: 606463). The predominant phenotype results in hepatosplenomegaly, bone marrow dysfunction resulting in thrombocytopenia, anaemia and bone disease. Accumulation of substrate in other tissues such as the liver or lungs can result in additional disease morbidity, patients with disease isolated to these systemic tissues are considered to have non-neuronopathic ‘type 1’ Gaucher disease. A clinically more severely effect group of patients have substrate accumulation in the CNS and their disease-course is variable. Those with a rapidly progressive form in the neonatal period and who die within the first 2 years of life have ‘type 2’ acute neuronopathic disease and those with a slower, more progressive neurological phenotype have been termed ‘type 3’ or ‘chronic neuronopathic’. ‘Neuronopathic Gaucher disease’ has been defined as Gaucher disease with neurological signs or symptoms which cannot be attributed to any other pathology [1] and, more recently, refined by an expert consensus group to be a biochemical and genetic Gaucher disease with the clinical finding of a gaze palsy [2]. A notable exception to the definition are those patients with Gaucher-related Parkinson’s Disease (PD) in whom there is clearly neurological feature (PD) however the primary mechanism is associated with *GBA1* mutation rather than CNS Gaucher cell accumulation.

In the early 1990s treatment for Gaucher Disease was established [3], enzyme replacement therapy (ERT) revolutionised the lives of patients, halting the progression of established systemic disease features and reversing some. Insufficient and ineffective ERT penetration of the blood–brain barrier (BBB) means that central substrate accumulation persists in patients with neuronopathic phenotypes and the neuropathology, although demonstrated [4], remains poorly understood. The clinical features and course of CNS disease is markedly heterogenous.

In the 1970s, case reports identified a series of patients with neuronopathic disease who had saccadic eye movement abnormalities [5, 6] and it is now accepted that saccade initiation failure, followed by slowing of saccades and eventual saccadic palsy are universal clinical features of neuronopathic disease [1, 7]. The consistency of this finding has, to date, been limited by the difficulty in measuring eye movements in clinical settings. Furthermore, being able to clinically detect them is a skill requiring experience, particularly in children. Subtle compensatory techniques are often adopted by children, which confirm the presence of the pathology; these include excessive blinking to initiate saccades or ‘head thrusting’ to aid in moving the eyes towards the target [8].

The relationship between horizontal saccade abnormalities and the neuropathology of nGD is not well understood. In mouse models of disease, focal microglial activation in the area of the Substantia Nigra Reticulata and the Reticulotegmental Nucleus of the Pons (Nucleus of Bechterew) [9] has been demonstrated. The generation of saccades is within the Paramedian Pontine Reticular Formation (PPRF), part of the reticular formation which runs parallel to the pontine nucleus and extends throughout the brainstem [10]. It is likely that that pathology in this area; as demonstrated by Wong et al. [4] is responsible for the clinical sign but the vulnerability of this region to the disease process has not been explained.

Normal saccades are fast, *generally* voluntary, conjugate eye movements which enable rapid alteration of fixation [11]. They are best exemplified by the movement of the eyes while reading. Measures of saccadic movement include velocity, gain (a measure of accuracy in generating a desired amplitude of saccade in response to target), latency (the time taken to initiate a saccade in response to the appearance of the target) and duration. Historical methods of measuring oculomotor function have included the invasive, scleral search coil technique [12], infrared light methods [13], video-oculography (VOG) and electrooculography (EOG). EOG involves application of electrodes to skin surrounding the eyes and detects eye movements through electrical impulses generated by eye movements, this method although less invasive, is less accurate and vulnerable to artefact from muscle tension underlying the electrodes [14]. Contemporary video-oculography offers a method which is acceptable to patients and is increasingly being demonstrated to show consistent accuracy.

Interest in saccadic eye movements in Gaucher disease has gained increasing attention as our understanding of Gaucher disease is changing. The aforementioned unexplained relationship between variants of *GBA1* and Parkinson’s Disease [15] has encouraged further evaluation of the spectrum of Gaucher phenotypes. Furthermore, as more detailed phenotyping is undertaken and increasing numbers of international cohorts of patients are being described, the spectrum of neurological involvement in Gaucher disease is expanding [16].

The greatest interest in oculomotor function in Gaucher disease is the pursuit of quantifiable outcome measures for therapeutic trials for neuronopathic disease. Given the variability in clinical features and rate of disease progression, a good biomarker is required to demonstrate efficacy of novel drugs. To date, no robust biochemical or clinical measure has been identified. As saccadic function is impaired in all patients with nGD, it is the single clinical feature which has potential to be measured and which has been previously used as measure in interventional trials [17].

Here we report on the experience in the UK of measuring saccadic eye movements using video-oculography (EyeSeeCam) in Gaucher Patients with type 1 and type 3 disease. We evaluated 60 patients and 29 healthy controls with a view to replicating the previously reported slowing of saccadic movements in patients with neuronopathic disease [18], evaluating the role of longitudinal analysis for disease monitoring and outcome measure development and examining the role of such a device in supporting diagnosis of neuronopathic disease.

## Methods

### Participants

A total of 60 Patients with Gaucher disease were recruited from seven specialist centres in the UK and underwent video-oculographic assessment (for details of the protocols, see Recordings of eye movements). For inclusion, patients were at least five years of age at enrolment and had a biochemical and genetic diagnosis of Gaucher disease [19, 20]. All participants consented to study procedures according to full ethical approvals (UK research ethics committee approval 16/WA/0129; Wales NHS REC Bangor; IRAS 192163) and provided their written and informed consent.

Although examination was undertaken in 21 patients with type 3 Gaucher disease, only 14 were adequate for comprehensive analysis; similarly, 39 patients with GD-T1 were examined and only 36 were suitable for inclusion (total analysed Gaucher cohort; *n* = 50). Analysis was made difficult in the presence of significant strabismus, excessive compensatory blinking or such profound palsy that no saccadic movement was recordable clinically. The patients with nGD excluded from comprehensive analysis had the most profound saccadic impairments.

The Gaucher cohort (*n* = 50, median age 32 years, range 5–77 years) comprised two groups of patients that were included in the final statistical analysis: (GD-T1) Patients with a clinical diagnosis of ‘type 1’ Gaucher disease (*n* = 36, age 38 years, range 5–77 years), and (nGD) patients with ‘type 3’ Gaucher disease (*n* = 14, age 23 years, range 17–33 years). The patient classification was provided by the clinician caring for the patient (all experts in LSD management) and was consistent with the traditional phenotypic approach to categorisation of GD-T1 as Gaucher disease ‘with lack of early onset central nervous system (CNS) involvement’ [2]. Diagnosis of nGD typically requires the presence of a saccadic gaze palsy as the earliest manifestation of CNS involvement in GD with subsequent development of other neurological features, given the expertise of the clinical teams caring for these patients the clinical categorisation used to manage the patients care was adopted for the purposes of this study. Patient screening for the study consisted of confirmation of biochemical and genetic diagnosis of Gaucher disease; the clinical examination and phenotypic categorisation was not part of the screening process.

For comparison, healthy controls (*n* = 35, age 40 years, range 23–59 years) were recruited from a single research centre. None of the healthy volunteers had any clinically significant medical or psychiatric condition. All participants underwent a clinical examination of saccadic eye movements prior to video-oculographic recordings in order to define the eye providing the ‘better quality’ in the presence of strabismus or palsy. Any clinically detectable (on examination) abnormality of saccadic movements was recorded during this time and, as later discussed, had not been previously reported for some patients in the GD-T1 group by the caring clinician at time of screening; they therefore remained in the disease type group for the duration of the study, no reclassification of disease was made during the study period.

### Recording of eye movements

Measurements took place in an acoustically shielded and softly lit environment. Participants were seated with their head stabilised by an adjustable chin rest facing a specially dedicated computer screen at an eye-to-screen distance of *d* = 60 cm. Some children did not tolerate the chin rest and stabilised their heads with their hands. Monocular eye movements were video-oculographically recorded using the headmounted EyeSeeCam® device (EyeSeeTec GmbH, Munich, Germany) operating at temporal sampling rate of 220 Hz [21]. The room lighting was dimmed to maximise both patient’ focus and video recording of the pupillary motion. Patients were instructed to keep their head as motionless as possible and attentive focus on the series of target spot motion on the screen.

The stimulus protocol was adapted from Bremova-Ertl and colleagues [18] and comprised a calibration sequence followed by visually-guided reflexive prosaccades. All participants were instructed to re-fixate to the new target spot as rapidly and as accurately as possible and to withhold any unwanted gaze shifts. Verbal encouragement was offered to track the target motion and to minimize head movement and blinking where possible.

Prosaccades were pseudo-randomly elicited in vertical and horizontal direction by presenting target spots (*d* = 1.33°) at the screen. Vertical saccades were elicited by target steps of ± 10° and ± 20° within a target range of ± 10° with respect to the central position. Horizontal saccades were elicited by target steps of ± 15° and ± 30° within a target range of ± 15° with respect to the central position. Targets for both vertical and horizontal direction were presented for a pseudo-random duration ranging from 2.5 to 3.0 s. Each target step was repeated seven times in order to increase the probability of measuring a number of saccades that is sufficient for statistical analysis in patients with difficulties in performing saccades. The overall protocol lasted at most five minutes.

### Analysis of eye movements

An interactive MATLAB®-based software package as shipped with the EyeSeeCam® device was used for the analysis of eye movement traces. Prior to event detection in the recordings, noise reduction, deletion of artefacts such as blinks was performed. The recordings obtained from the calibration sequence was used to map the ‘raw’ signal to the ‘true’ orthogonalized eye position. An acceleration-based saccade detection algorithm for search coil data was used and adapted to automatically extract (1) saccade amplitude, (2) saccade peak velocity, (3) duration, and (4) reaction time from the eye tracker data with respect to the stimulus amplitude. All cases were visually inspected for proper saccade detection. In case for incorrect automatic saccade detection, the onset and offset of the respective saccades was manually performed. Slow saccades that did not meet detection criteria were manually selected.

### Saccade performance parameters

Saccade performance is expressed in four saccade parameters including (1) reaction time, (2) saccade duration, (3) peak saccade velocity, and (4) saccade gain for each stimulus direction, i.e. left, right, up, and down resulting in a total of 4 × 4 = 16 parameters.

The *reaction time* is almost independent of the saccade trajectory properties (duration, velocity, etc.) and is therefore provided as the average value.

The *saccade duration D* increases as a function of eye amplitude *A* and can be adequately modelled within a restricted range for 5° < *A* < 50° as

$$D\left( A \right) = D_{0} + k \cdot A$$

with linear model parameters *D*_0_ and *k* to be estimated for each individual [22].

*Peak saccade velocity* increases nonlinearly as a function of saccade amplitude. The exponential relationship

$$V_{{{\text{peak}}}} \left( A \right) = V_{\max } \left( {1 - e^{{ - \frac{A}{{A_{c} }}}} } \right)$$

produces very satisfactory fits [23] where *A*_C_ denotes an individual amplitude constant and *V*_max_ indicates the individual saturation velocity at large amplitudes ($A \to \infty$).

Saccades are frequently dysmetric and commonly undershoot the target for amplitudes *A* > 10°. The mismatch between Target *T* and saccade amplitude *A* can be expressed as the error amplitude $\epsilon = T - A$ which is an approximately linear function of the target distance:

$$\epsilon \left( T \right) = d \cdot \left( {T - T_{0} } \right)$$

The slope *d* is the rate of error amplitude increase and *T*_0_ a ‘neutral’ target distance were saccades are on average hit the target [22]; both parameters vary across individuals. Targets T > T0 commonly undershoot the target. A common measure of target-saccade amplitude mismatch is the *saccade gain G* which is the proportion between saccade amplitude *A* and target distance *T* and can therefore be expressed as a function of the error amplitude as follows:

$$G\left( T \right) = 1 - \frac{\varepsilon \left( T \right)}{T}$$

All saccade trajectory characterising parameters, i.e. saccade duration, saccade peak eye velocity, and saccade gain, are computed for each individual and stimulus direction as the readout from the respective fit at 20° and finally subjected to the statistical analysis (see Statistical Analysis section below).

### Computing reference ranges for healthy controls

Reference ranges were computed from the healthy control cohort (*n* = 29) in order to quantitatively appreciate the VOG measurement in the patient cohorts. The 95% prediction intervals (PI) were calculated by assuming a normal distribution for the respective parameter as follows:

$$PI_{95\% } = mean \pm t_{0.975,n - 1} \cdot sd \cdot \sqrt {\left( {n + 1} \right)/n}$$

### Statistical analysis

SPSS 23 (Version 23.0.0.0, 2015; IBM Corporation, Armonk, New York) was used for statistical data analysis. Data on participants’ demographic features and eye movement parameters were provided as median (interquartile range). Non-parametric inference statistics were used for hypothesis testing between groups as the values for patients’ groups cannot be assumed to be normally distributed. Fisher’s exact test was applied for categorical variables and Kruskal–Wallis analysis of variances or Wilcoxon- Mann–Whitney-*U*-test on ranks for continuous variables. In case of comparing three or more groups, Kruskal–Wallis analysis of variances on ranks was followed in the event of significance (*p* < 0.05) by Dunn’s multiple comparison test for pairwise post-hoc contrasts. Spearman's rank order correlation coefficient was used to describe the relationships between different scores. All statistical tests were 2-sided with *p* < 0.05 indicating statistical significance; *p* values were adjusted for multiple testing using family-wise error correction when contrasts were not driven by a specific hypothesis.

## Results

### Reference ranges from healthy controls

Out of a total of 35 healthy controls (HC), data was considered suitable for the final statistical analysis in 29. In those who it was considered ‘unsuitable’ this was a result of poor or incomplete saccade recordings caused by eye makeup which generated excessive artefact, participants falling asleep during the task, significant head movement artefact or technical failures of the recording.

Saccade parameters for left and right (mean values arithmetically averaged) were not statistically related to age as indicated by Spearman rank order correlations with (1) peak saccade velocity as obtained by a readout from the non-linear fit of the main sequence for an amplitude of 30° (*ϱ* = -0.04; *p* = 0.86), (2) mean saccade gain (*ϱ* = -0.08; *p* = 0.71), (3) mean saccade duration (*ϱ* = -0.19; *p* = 0.37), and (4) response time (*ϱ* = 0.40; *p* = 0.059). It is of note, that correlation between age and response time revealed a clear (but not significant) trend for increasing response time across the lifespan.

**Fig. 1:**
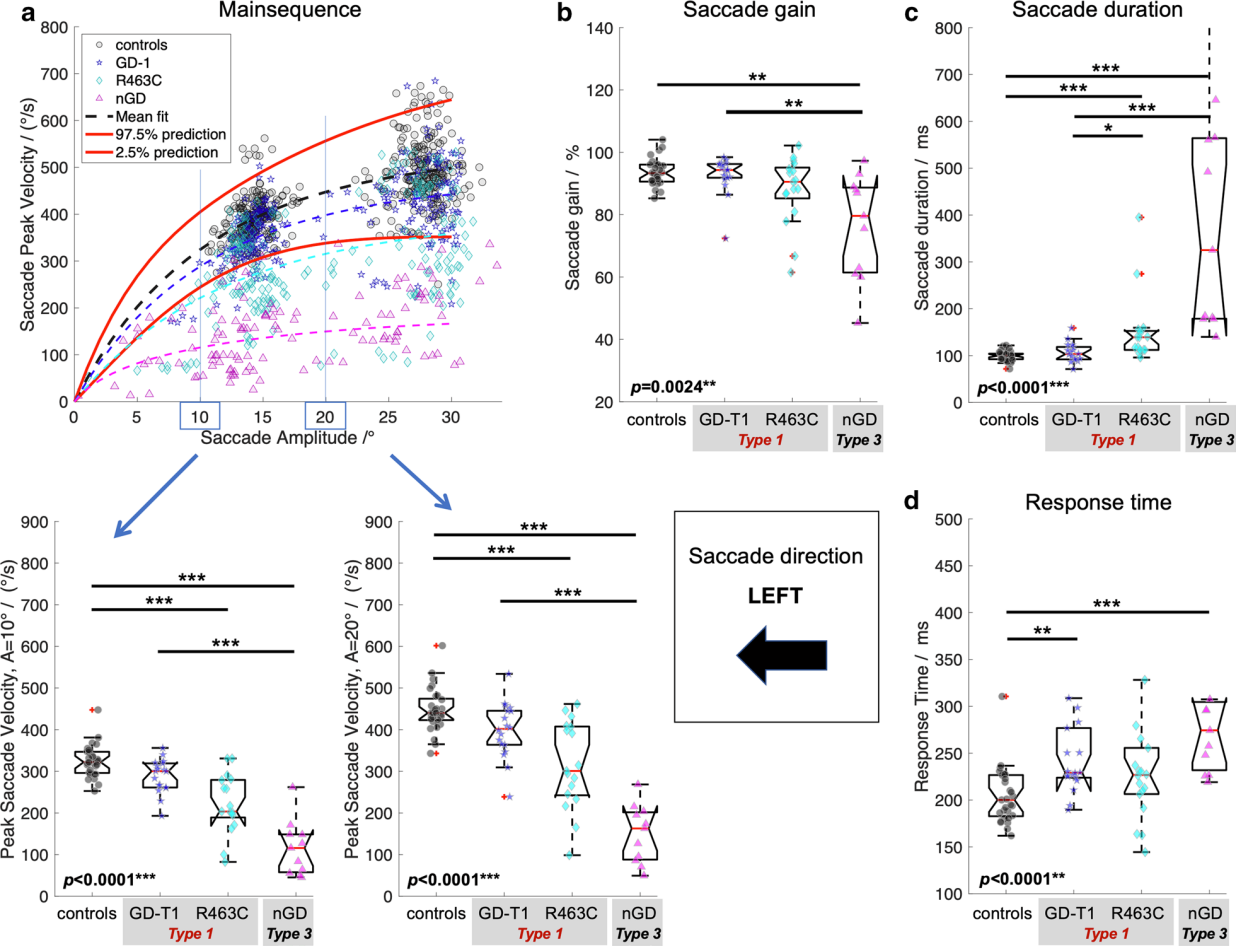
Saccade performance left.** a** Main sequence with individual’s data points and 95% prediction interval (red solid lines) with statistics of peak saccade velocities obtained from readouts at given eye amplitudes (left and right lower panel) from the individual’s non-linear (*V*_*ma*x-_*V*_max_*exp(-*A*/*A*_*c*_)) fit along the main sequence. **b** Saccade gain computed as the ratio of saccade amplitude and target amplitude. **c** Time duration from saccade onset to saccade end. **d** Response time as the time difference from target onset to saccade onset. Provided *p*-values resulted from Kruskal–Wallis analysis on ranks across groups, i.e. controls, Gaucher disease type 1 (GD-T1), Gaucher disease type 1 with R463C mutation (R463C), and Gaucher Disease type 3 (NGD). Statistically significant differences of post-hoc Dunn’s test are indicated by *p < 0.05; **p < 0.01; ***p < 0.001. All *p*-values are adjusted for multiple comparisons using the family-wise error rate

**Fig. 2:**
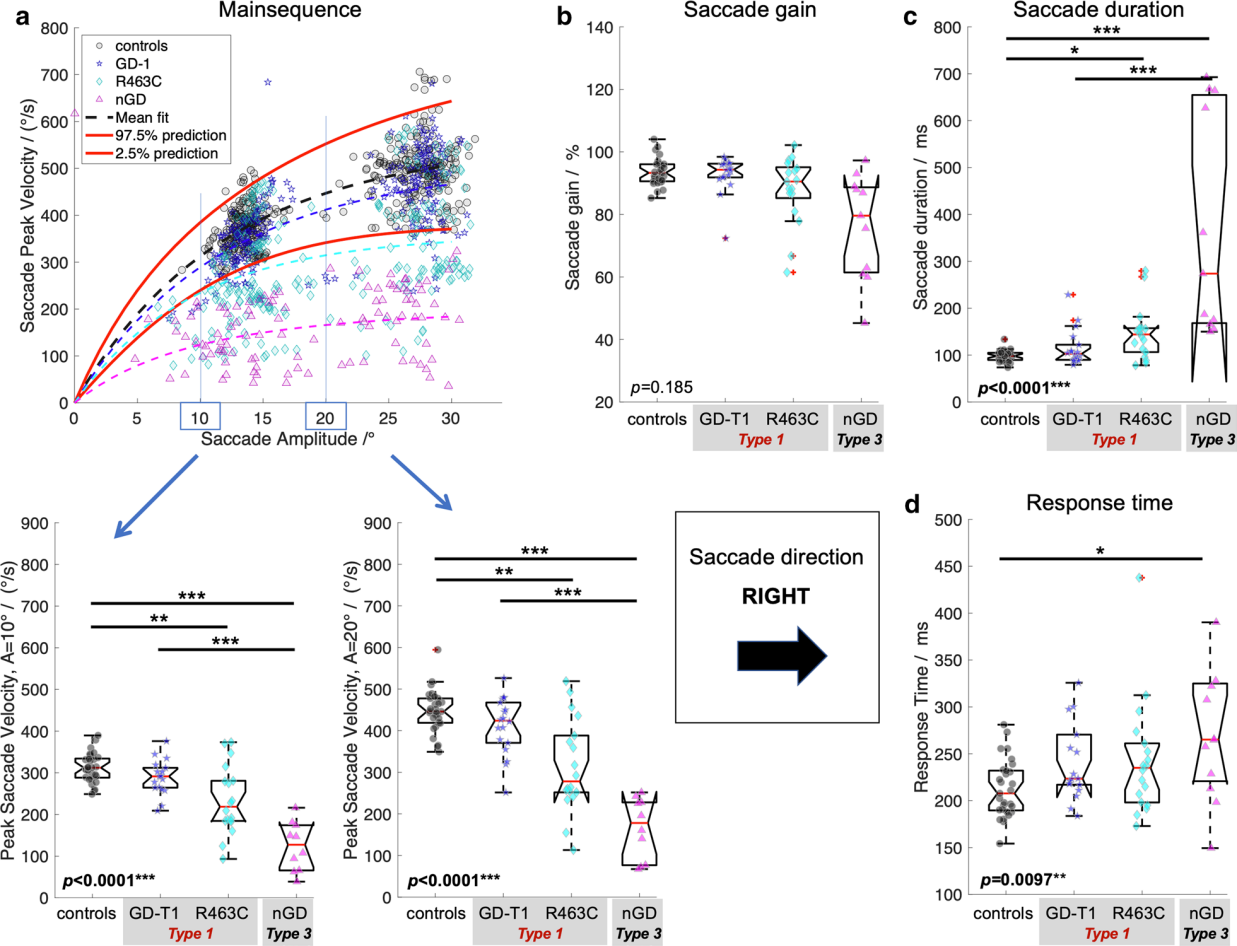
Saccade performance right.** a** Main sequence, **b** saccade gain, **c** time duration, and **d** response time. See Caption Fig. 1 for details

**Fig. 3:**
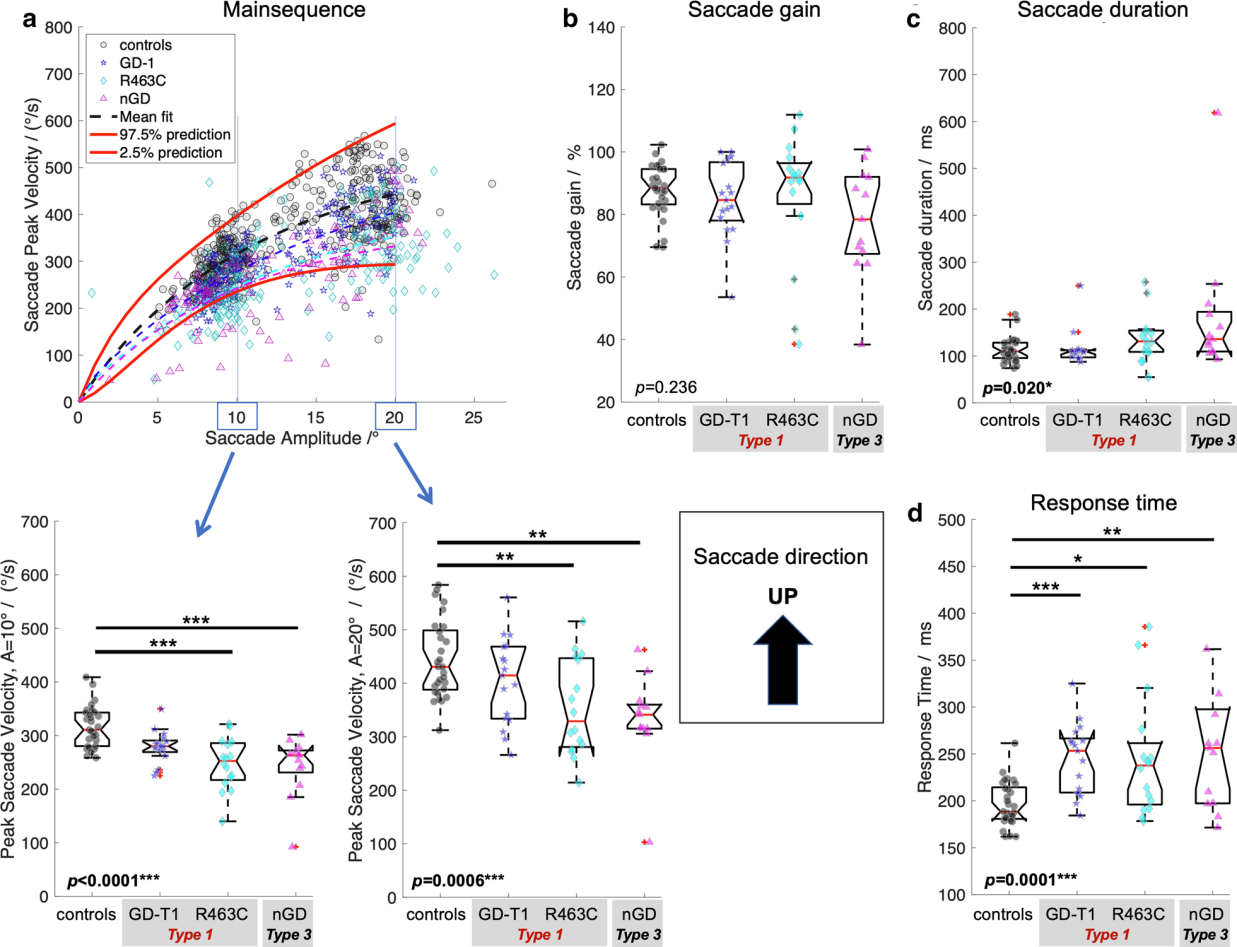
Saccade performance up.** a** Main sequence, **b** saccade gain, **c** time duration, and **d** response time. See Caption Fig. 1 for details

**Fig. 4:**
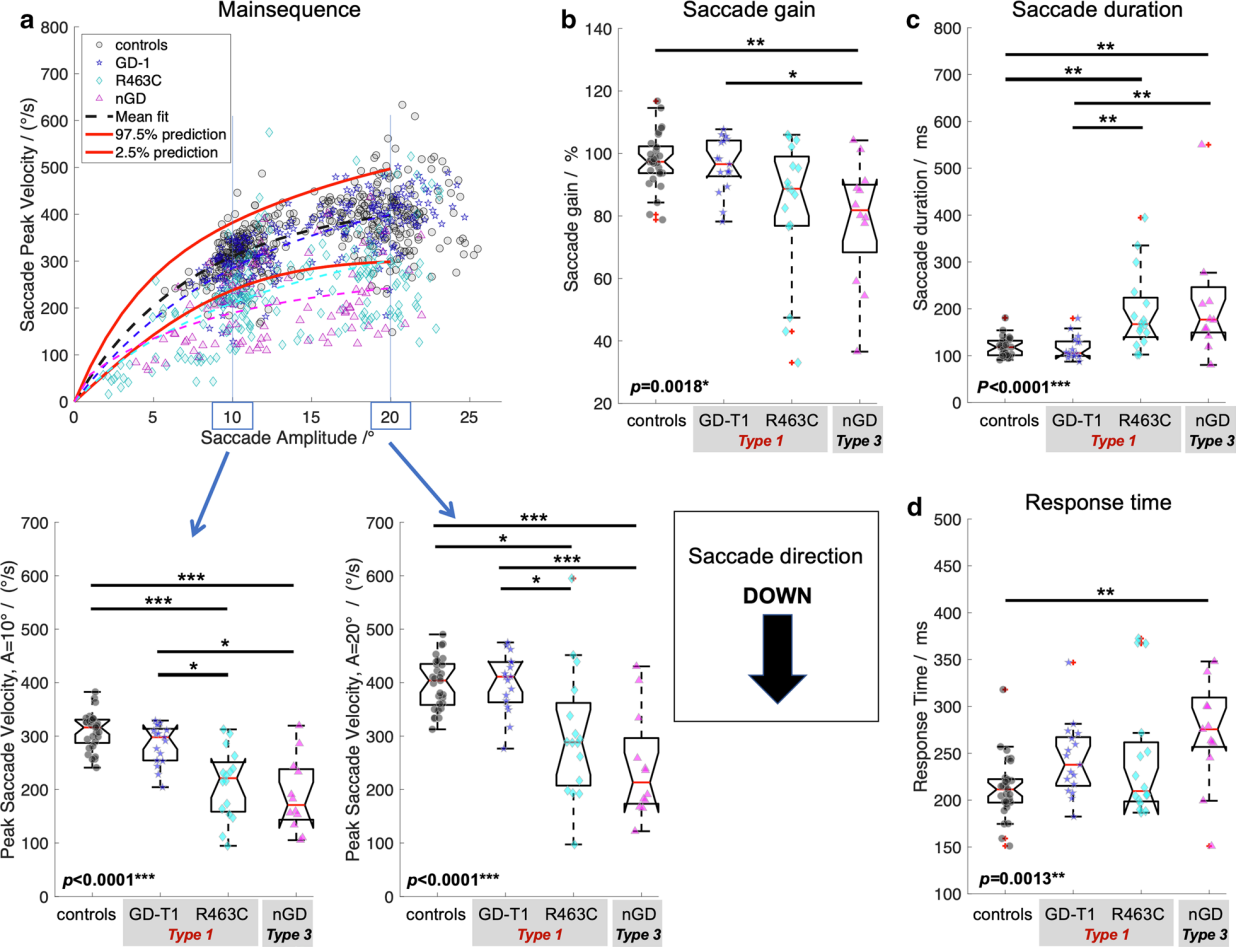
Saccade performance down. **a** Main sequence, **b** saccade gain, **c** time duration, and **d** response time. See Caption Fig. 1 for details

In order to investigate saccade performance in Gaucher disease, reference ranges for controls were computed. Table 1 summarises the reference ranges (95% prediction interval) for the control cohort (*n* = 29) for each of the investigated oculomotor parameters. In addition, Figs. 1a–4a show the reference range for the main sequence which was obtained by computing the reference range for readouts at given amplitudes (i.e., 0°, 1°,2°, …) from the individual’s non-linear (*V*_*ma*x-_*V*_max_*exp(−*A*/*A*_*c*_)) fit along their main sequence.

**Table 1:** Reference ranges

	Mean value	Reference range
Response time^a^		
Left/ms	205	144–267
Right/ms	213	149–277
Up/ms	197	147–246
Down/ms	213	145–281
Saccade duration^b^		
Left/%	99	76–122
Right/%	98	74–123
Up/%	113	58–167
Down/%	118	75–161
Saccade peak velocity^c^		
Left (10°)/(°/s)	325	244–405
Left (20°)/(°/s)	448	338–556
Right (10°)/(°/s)	312	240–385
Right (20°)/(°/s)	447	342–552
Up (10°)/(°/s)	316	236–397
Up (20°)/(°/s)	443	293–594
Down (10°)/(°/s)	310	238–382
Down (20°)/(°/s)	398	299–497
Saccade gain^d^		
Left/%	94	85–103
Right/%	90	80–101
Up/%	88	70–105
Down/%	97	80–116

Data are provided as the population mean and the 95% prediction interval as computed for the healthy control cohort (*n* = 29)

^a^Time duration from target onset to saccade onset

^b^Time duration from saccade onset to saccade end obtained from readouts of the linear amplitude—saccade duration fit at 20° for each individual

^c^Peak saccade velocity obtained from readouts at 10° and 20° from the individual’s fit along the main sequence

^d^Ratio of saccade amplitude and target amplitude obtained from readouts of the target distance—gain fit at 20° for each individual

### Quantitative analysis of saccade performance in Gaucher disease

Saccade performance in patients with Gaucher disease (Type 1 and Type 3) was compromised in all aspects, i.e. response time, saccadic duration, peak eye velocity, and saccadic gain.

Compared to controls, nGD patients presented considerably longer response times in all directions (Kruskal–Wallis *p* < 0.0035, post-hoc *p* < 0.0051, family-wise error corrected) whereas response times between Gaucher subtype 1 and 3 did not statistically differ (post-hoc *p* > 0.124). GD-T1 patients presented longer response times than controls, an effect that was significant for leftwards (post-hoc *p* = 0.0044) and upwards (post-hoc *p* < 0.0001) gaze.

Saccade duration was statistically different across groups (Kruskal–Wallis *p* < 0.038). Saccades in nGD vs. controls were significantly prolonged in all directions (post hoc *p* < 0.031, family-wise error corrected). Post-hoc testing for nGD vs GD-T1 revealed prolonged saccade duration for horizonal saccades (*p* < 0.0007) but not in vertical direction (*p* > 0.099). Post-hoc testing indicated prolonged saccades between GD-T1 and controls for horizonal (*p* < 0.032) but not for vertical saccades (*p* = 0.585).

As expected, main sequence analysis of peak saccade velocity readout from the main sequence function at the amplitude of 20° indicated a significant difference between nGD (Type 3) and controls (Kruskal–Wallis *p* < 0.0007, post-hoc Dunn’s test, *p* < 0.0013). Surprisingly, however, there was a significant difference in peak saccade velocity also between GD-T1 and controls (post-hoc, *p* < 0.017, family-wise error corrected), with the exception of downward peak velocities (*p* = 0.267).

Saccade gain analysis revealed hypometric saccades in nGD vs. controls (Kruskal–Wallis *p* < 0.034, post-hoc leftwards and downwards, p < 0.0025, family-wise error corrected), but was similar in GD-T1 and controls (*p* > 0.123).

### Subgroup saccade analysis in type 1 Gaucher disease (GD-T1)

To further investigate the difference in patients with type 1 Gaucher disease relative to controls (which was not expected), their peak eye velocities were compared with the reference range (for definition see below) at the individual level. Abnormal peak saccade velocities (velocities below the reference range according to Table 1) were demonstrated in 19 (56%) patients with type 1 Gaucher disease (of *n* = 36). I n five patients the velocity was slowed in two or less measures of vertical gaze, which is less specifically relevant to saccade abnormalities in Gaucher disease. An examination of the clinical characteristics of the fourteen GD-T1 patients who had abnormality of saccade velocities in three or more measures was therefore undertaken with a view to identifying unifying features; see Table 2.

**Table 2:** Clinical characteristics of fourteen patients with GD-T1 with abnormality of saccadic velocity in three or more measures

Age	Age Dx	Age at ERT	Genotype	Spleen	Gaucher related Co-morbidities	Number of velocity measures abnormal	Direction of abnormality	Clinical saccade abnormality
53	5	32	R463C/RecNcil	S	Liver disease	4	Left & right	Y
49	19	37	R463C/RecNcil	S		6	All	Y
55	47	47	R262G/RecNcil		Abnormal neurology	4	Left, right, down	Y
48	4	25	R463C/IVS2+1	S	Liver disease & Lung disease	6	Left, right, down	Y
77	56	57	R463C/L444P		Lung disease	6	Left, right, up	Y
15	8	8	R463C/R257Q			8	All	
70	6	54	R463C/G377R	S	Lung disease	5	All	Y
64	6	46	R463C/RecNcil	S	Cognitive Impairment	6	Left, right, down	Y
18	3	3	R463C/N462K			4	Left & down	
73	48	56	R463C/L444P	S		6	All	Y
31	5	6	R463C/L444P		Liver disease & subtle ataxia	8	All	Y
43	2	27	R463C/L444P	S		8	All	
16	3	3	R463C/RecNcil			3	Right & down	
12	11mo	1	H311R/R359Q		Liver disease; lung disease & lymphadenopathy	4	Left & right	Y

Dx: Diagnosis; Age given in years; Genotype: Traditional GBA1 variant nomenclature used; R463C (p.Arg502Cys); RecNcil (recombinant consisting of multiple pseudo-gene derived point mutations); L444P (p.Leu483Pro); IVS2+1 (Splice site variant c.115+1G > A); G377R (p.Gly416Arg); R262G (p.Arg301Gly); R257Q (p.Arg296Gln); N462K (p.Asn501Lys)

S, splenectomised; Y, yes/present

^*^Genotype documented but not confirmed

The striking shared feature is presence of the *GBA1* variant p.Arg502Cys (c.1504C > T; traditional nomenclature: R463C) in 12 of 14 of these patients. When correlated with clinical examination, a very subtle defect of eye movements (clinical saccade slowing or delayed initiation) was identifiable in most patients.

The identification of a fourth subgroup (Gaucher Type 1 with an R463C mutation) enabled us to refine the cohorts and re-perform the statistical analysis for saccade parameters across all groups. The Gaucher cohort (*n* = 50) comprised three groups of patients that were included in a second statistical analysis: GD-T1; patients with a clinical diagnosis of ‘type 1’ Gaucher disease without R463C mutation (*n* = 18); R463C patients with type 1 Gaucher disease and a single allele with an R463C mutation (*n* = 18) and (nGD) patients with ‘type 3’ Gaucher disease (*n* = 14). Saccade performance in patients with Gaucher disease with the refined subgroups is comprehensively summarized in Fig. 1–4. Kruskal–Wallis analysis on ranks for mean saccadic eye movement parameters (i.e., peak eye velocity, saccadic gain, saccadic duration, and response time) across these four groups (GD-T1, R463C, nGD, controls) revealed statistically significant differences for each parameter in left (Fig. 1), right (Fig. 2), up (Fig. 3), and down (Fig. 4) direction (*p* < 0.0043) with an exception of right saccade gain (Fig. 2b) and response time (Fig. 2d), and upward saccade gain (Fig. 3b). Relative to controls, post-hoc testing using Dunn’s test followed by family wise error correction revealed that saccade performance was impaired in all patient groups (reduced peak velocities, hypometric saccades, prolonged saccade duration and response times).

## Discussion

This study was undertaken to evaluate the use of video-oculography as a tool to measure saccadic eye movement parameters in Gaucher Disease. We have replicated the previously reported data showing lower peak velocity in horizontal and vertical saccadic eye movements in nGD patients versus a control cohort [13, 18, 24, 25]; although the specific values in the cohort are slightly higher than those reported by Bremova-Ertl et al. [18] the difference between the values is consistent. The ability to replicate data findings between disease states is supportive of a role for such a device for clinical use. However, the lack of a large cohort study showing normative values in healthy controls is a limitation of implementation.

Saccadic eye movements in the context of Gaucher disease are becoming increasingly important. Defects in saccade initiation are thought to be the earliest sign of CNS involvement in Gaucher disease and are eagerly pursued at time of diagnosis to help offer patients and families prognosis. However, as we enter an era in Gaucher disease with increasing treatment options and clinical trials, determination of disease categorisation is becoming ever more important [26, 27]. Therapeutic strategy and eligibility for interventional trials is determined, in part, by which disease ‘type’ a patient has been categorised as.

In this series we have been able to show, using this non-invasive quick test and measuring just a few parameters, that patients with different disease types (and now also different genotypes) can be differentiated on the basis of saccadic parameters. However, we also were *unable* to report objectively on the saccadic movements of several patients with known, profound defects. Furthermore, we only successfully examined six patients aged 12 years and younger; the youngest two patients were aged five years at time of recording, one with type 1 disease and one with type 3 disease; a six-year-old with type 3 disease also underwent examination but the quality of the recordings (due to severity of abnormality) limited detailed analysis. A further two patients with profound type 3 disease weren’t approached for examination in view of their difficulties in cooperating with instructions and ability to remain still for the duration of testing. This inability to use the EyeSeeCam in all settings raises questions about its utility in diagnosing saccadic eye movement problems. However, for those with profound deficits or profound Gaucher-related neurology which prevents examination, there generally is no diagnostic uncertainty, therefore the role for the EyeSeeCam in confirming diagnostic category is with the patients in whom the clinical findings are subtle or equivocal, typically a group of patients who are able to cooperate and tolerate this examination.

The most striking finding in this study was a cohort of patients who had been phenotypically categorised as having type 1 Gaucher disease but who have significantly slower saccadic eye movements than healthy controls and generally more severe systemic phenotypes than would be expected of type 1 Gaucher disease. Some such patients also had subtle additional neurological features which hadn’t previously been explained. Although these findings were all subtle and hadn’t provoked the attention of their caring physician (which suggests minimal functional impact), it prompts consideration of each individual’s future prognosis which in the current environment of pursuit of CNS-penetrant therapy they may be excellent candidates for. We also have not examined the saccadic eye movements of patients with Parkinson’s disease in this study or patients with Gaucher-related Parkinson’s disease, the neuropathophysiology of which, remains elusive. Slowing of saccades may be indicative of such evolving pathology [28]. Many historical reports exist of varying neurological features in type 1 Gaucher disease which have yet to be truly untangled in their relationship to both Gaucher disease and Gaucher-related Parkinson’s disease [29–33].

The unifying feature of these patients with GD-T1 and saccadic abnormalities, was the presence of a *GBA1* variant, p.Arg502Cys (R463C), most frequently seen here associated with a second ‘severe’ or previously described ‘neuronopathic’ variant on the opposing allele. This variant has been previously implicated in neuronopathic disease and specifically in the development of saccadic slowing [34]. R463C is also present in three of the patients in the type 3 cohort examined, all who had an adult diagnosis of type 3 disease. Although in many other rare diseases, clinical assessment of phenotype has been superseded by genotype evaluation; genotype: phenotype correlations in Gaucher disease is incomplete and continues to be disputed. This in part reflects the vast number of variants (> 400 reported to date) but also the marked phenotypic heterogeneity displayed. it is thought that multiple environmental and genetic modifiers are implicated in explaining the spectrum of disease encountered [30, 35–37]. Only very few *GBA1* pathogenic mutations have shown consistent phenotype correlation [38] and therefore clinical evaluation remains the primary source of such prediction. As more detailed methods of characterising the *GBA1* variants in the disease are established, larger cohorts are reported, and more detailed longitudinal phenotyping is undertaken, we may see that a greater relationship between genotype and phenotype exist which offers opportunities for therapeutic stratification.

The study also aimed to evaluate the role of the EyeSeeCam as an outcome measure for clinical and trial purposes. Correlation with markers of disease severity in this setting is particularly difficult, given the lack of other robust biomarker of neuronopathic disease. The modified Severity Scoring Tool (mSST) [39] was designed and implemented as a clinical tool for this purpose and has shown utility, however a component of the tool includes saccadic eye movement deficits and the cohort presented here with objective measures with neuronopathic disease was small. Correlation has been demonstrated with vertical saccade duration and mSST previously [18, 24] but a larger cohort study is required and ideally a more detailed scoring tool or biomarker to demonstrate CNS involvement.

### Limitations

A larger cohort of control data confirming any subtle differences in saccade parameters by age would be of value. Observations from this cohort of controls may not have been adequately powered to demonstrate significant statistical difference by age; however previous studies (using various devices to measure saccadic movements) have shown that with increasing age, peak saccade velocity is reduced [40]. Saccade latency is also determined partly by specific areas in the cerebral cortex and is therefore vulnerable to greater variability, even in children, latency changes with age in some studies [41]. The lack of Paediatric controls is a limitation to interpretation, previous studies have shown that saccade velocities are stable throughout the paediatric age groups and match those of adult cohorts [41, 42]. The saccadic slowing identified in the GD-T1 group however would not be thought to reflect age-related saccadic changes given the significant number of young adults they were found in. Other potential confounders; fatigue, caffeine intake or mental health diagnoses which may affect oculomotor function were not controlled for in either healthy controls or disease groups; these should be considered in future studies as they have potential to impact oculomotor parameters [43, 44].

## Conclusion

This study showed that a subgroup of patients with type 1 Gaucher disease and a shared *GBA1* mutation all had significantly slowed saccades suggestive of a greater phenotypic spectrum of Gaucher disease than previously described.

Video-oculography devices such as the EyeSeeCam have utility in objectively measuring eye movements in patients with mild to moderate defects or in cases where clinical examination is inconclusive. This is useful in the context of Gaucher disease, where presence of oculomotor abnormalities determines disease categorisation and may indicate prognostic differences and therefore alter therapeutic strategies. Given the rarity of the disease and delays in expert assessment, objective methods of measuring consistent clinical features to support such categorisation is essential and even greater in the setting of interventional clinical trials; larger datasets are needed to establish this as part of standard clinical assessments and to define the pathological thresholds of the various oculomotor parameters.

This study has highlighted a broader spectrum of type 3 Gaucher disease, indicating that features of neuronopathic disease may not be discernible until later in life, future studies and evaluation of these patients over time will aid in understanding the clinical relevance of this for these patients. These findings also highlight the need for repeated clinical assessment of patients following diagnosis. Although not a focus of the reported data, the high burden of non-neurological Gaucher disease complications in the GD-1 R463C cohort was noted and may reflect the greater systemic disease experienced by patients with nGD. Furthermore, these observations raise the question of the utility of current phenotypic categorisation of Gaucher disease for patients where the disease descriptions are becoming increasingly heterogeneous.

## Data Availability

The datasets used and/or analysed during the current study are available from the corresponding author on reasonable request.

## Abbreviations

LSD: Lysosomal storage disorder
CNS: Central nervous system
ERT: Enzyme replacement therapy
BBB: Blood brain barrier
nGD: Neuronopathic Gaucher disease
VOG: Video-oculography
EOG: Electro oculography
HC: Healthy control
